# Ecological Stoichiometry and Density Responses of Plant-Arthropod Communities on Cormorant Nesting Islands

**DOI:** 10.1371/journal.pone.0061772

**Published:** 2013-04-23

**Authors:** Gundula S. Kolb, Cecilia Palmborg, Peter A. Hambäck

**Affiliations:** 1 Department of Ecology, Environment and Plant Sciences, Stockholm University, Stockholm, Sweden; 2 Department of Agricultural Research for Northern Sweden, Swedish University of Agricultural Sciences, Umeå, Sweden; University of Tartu, Estonia

## Abstract

Seabirds deposit large amounts of nutrient rich guano on their nesting islands. The increased nutrient availability strongly affects plants and consumers. Consumer response differs among taxonomic groups, but mechanisms causing these differences are poorly understood. Ecological stoichiometry might provide tools to understand these mechanisms. ES suggests that nutrient rich taxa are more likely to be nutrient limited than nutrient poorer taxa and are more favored under nutrient enrichment. Here, we quantified differences in the elemental composition of soil, plants, and consumers between islands with and without nesting cormorant colonies and tested predictions made based on ES by relating the elemental composition and the eventual mismatch between consumer and resource stoichiometry to observed density differences among the island categories. We found that nesting cormorants radically changed the soil nutrient content and thereby indirectly plant nutrient content and resource quality to herbivores. In contrast, consumers showed only small differences in their elemental composition among the island categories. While we cannot evaluate the cause of the apparent homeostasis of invertebrates without additional data, we can conclude that from the perspective of the next trophic level, there is no difference in diet quality (in terms of N and P content) between island categories. Thus, bottom-up effects seemed mainly be mediated via changes in resource quantity not quality. Despite a large potential trophic mismatch we were unable to observe any relation between the invertebrate stoichiometry and their density response to nesting cormorant colonies. We conclude that in our system stoichiometry is not a useful predictor of arthropod responses to variation in resource nutrient content. Furthermore, we found no strong evidence that resource quality was a prime determinant of invertebrate densities. Other factors like resource quantity, habitat structure and species interactions might be more important or masked stoichiometric effects.

## Introduction

Seabirds strongly affect the nutrient pools on their nesting islands by depositing huge amounts of nitrogen and phosphorus rich guano [1], [2]. They have been reported to increase plant nutrient content and primary productivity and to change plant species composition [3]–[5], but extremely high nest densities may lead to decrease primary production and vegetation cover due to ammonia poisoning [6], [7]. Such qualitative and quantitative changes in primary producers are likely to strongly affect higher trophic levels [8]–[10]. Not surprisingly, previous studies show large differences in the abundance of various invertebrate taxa when comparing seabird and non-seabird islands [7], [11]–[13], but it is unclear whether responses are mainly due to quality or quantity of resources. In this study we focus on the possibility that changes in resource quality might cause changes in consumer abundances.

Herbivores and plant feeding detritivores, especially, face the problem of a fundamental mismatch between the elemental composition of their body tissues and their resources; consequently nutrient limitation seem to be common in herbivore and detritivore populations [10], [12]–[14]. Predators may similarly be nutrient (nitrogen) limited due to imbalances in nutrient content between herbivorous and predatory insects [14]–[16]. Increased plant nutritional quality – as observed on seabird islands - can therefore both increase herbivore performance and density [10], [17]–[19] and raise predator and parasitoid performance and density [14], [20]–[25]. However, the mechanism underlying changes in predator and parasitoid performance and density in fertilization experiments is contested as changes in plant nutritional quality may or may not change the nutrient content of their consumers [23]–[26]. While plants often show high variability in nutrient content, due to an ability to store excess nutrients in the vacuoles, herbivores rather excrete excess nutrients and thereby maintain a stoichiometric homeostasis [27]. The generality of this strict stoichiometric homeostasis has recently been questioned and the results of several studies show that the C:N:P ratio of some heterotroph species varies with diet nutrient content (e.g., [10], [28], reviewed by [29]) or predation risk [30].

Predicting the effect of nutrient additions on the density of specific taxa is fraught with difficulties not only because of trophic feedbacks [31] but also because we lack a general theory on the connections between species traits, nutrient demand, and population growth. Plant ecologists have made several attempts to connect these processes, and a key finding is that competitive ability may be predicted from the nutrient use efficiency and the internal nutrient demand [32]. The fundamental role of individual nutrient demand in all ecological processes and interactions has been generalized in the development of ecological stoichiometry (ES, e.g., [27], [33], [34].

ES assumes that taxon-dependent differences in elemental composition determine differences in nutrient demand among species [27]. The growth rate hypothesis [35] suggests a mechanistic linkage between phosphorus (P) content and growth rate and reproduction of organisms; it states that differences in organismal C:N:P ratios are caused by differential allocation to RNA necessary to meet the protein synthesis demands of rapid growth rates [27]. Nutrient rich taxa are predicted to have a higher nutrient demand than nutrient poor taxa, and are therefore both more likely to be nutrient limited and more favored by nutrient enrichment than nutrient poor taxa [27], [36], [37]. We would thus predict that nutrient rich taxa should display a stronger positive density response on cormorant islands than nutrient poor taxa. This shift in species composition would also be reflected in an increased N:C and P:C ratio across species within taxonomic groups on or nearby cormorant islands. However, an elevated N:C or P:C ratio within a taxonomic group could be caused not only by a changed species composition but also by a deviation from strict homeostasis within a species.

In this study, we tested these possibilities on a set of islands with and without cormorant colonies in the Stockholm archipelago, Sweden. The effects of cormorant colonies on island vegetation, density and species composition of island arthropods, and near-shore algae and their associated invertebrates were reported in three previous studies [5], [7], [16]. Here, we first quantified changes in the soil nutrient composition among island types, to verify that cormorants had the predicted effects on soil N and P. Second, we estimated elemental composition of plants to determine whether changes in soil nutrient composition translated into changes in only plant biomass or also in nutrient content. Third, we investigated the pattern of body elemental composition of invertebrates and explored changes in elemental composition of several invertebrate taxa along the gradient of soil and plant nutrients. Thus, we investigate if bottom-up effects caused by cormorant nutrient input may be mediated by changes in resource quality. For the consumers we conducted our analysis mostly at the family or order levels since sufficient number of individuals could not be collected at the species level. Fourth, we tested if consumer density changes correlated with changes in resource quality (measured as leaf N:C and P:C) or quantity (measured as aboveground biomass) or with other vegetation characteristics (vegetation cover or plant species richness). Finally, we related the elemental composition of the investigated taxa and the elemental mismatch between their body tissue and their resource to their density response to nesting cormorant colonies.

## Materials and Methods

### Sampling

Sampling took place on and nearby islands in the Stockholm archipelago, Sweden (N 59° 20′ E 18° 03′) in summer 2007–2009. The archipelago consists of about 24 000 islands whose sizes vary between less than one m^2^ and several km^2^. Cormorants (*Phalacrocorax carbo*) recolonized the Stockholm archipelago in 1994 after hundreds of years of absence and increased strongly in numbers until 2007, when the population size appeared to have stabilized [38], [39]. Between April and August, the cormorants are largely confined to about 20 colonies spread across the archipelago, with a total of more than 5 200 nests, both on the ground and in trees [38], [39]. We sampled 25 islands: eleven active and three abandoned cormorant nesting islands and eleven reference islands without nesting cormorants. The active cormorant islands could be divided into islands with low and high nest density. On 12 islands we sampled both on land and in water, on seven only on land and on six only in water.

19 islands were used for terrestrial sampling; from these islands data about vegetation cover, aboveground plant biomass, plant species composition, arthropod densities and sampling design were available from former studies [5], [7]. Aboveground plant biomass showed a humped-shaped relationship to nest density, whereas vegetation cover and plant species richness were lower on active cormorant islands than reference islands and decreased with increasing nest density [5], [7]. On these islands we collected 2–6 common plant taxa (Poaceae, *Tanacetum vulgare*, Urtica dioica*, Alnus glutinosa, Sorbus aucuparia*, and Juniperus communis) and up to 20 arthropod groups belonging to three major trophic groups (taxa with * were only used for the comparison of CN among trophic groups): herbivores: Cercopidae (Auchenorrhyncha, Hemiptera), Heteroptera (Hemiptera)*, Aphidina, Lepidoptera larvae, Curculionidae (Coleoptera), and Chrysomelidae (Coleoptera); detritivores: Isopoda, Collembola, Brachycerid Diptera, Nitidulidae (Coleoptera)*; predators: Araneidae (Araneae), Linyphiidae (Araneae), Tetragnathidae (Araneae), Lycosidae (Araneae), Coccinellidae (Coleoptera), Carabidae, (Coleoptera), Staphylinidae (Coleoptera); *Nabis* spp. (Nabidae, Heteroptera), *Chartoscirta elegantula* (Saldidae, Heteroptera), Chilopoda*, and Formicidae; and Chironomidae (Diptera, midges with aquatic larvae). Chironomidae were not sorted into trophic groups and are therefore treated separately, but the dominating group in the area are detritivores. Arthropods were sampled with a converted leaf blower (Stihl® BG85 Leaf Blower/VAC), a sweeping net and by hand. Collected arthropods were transferred into plastic tubes and frozen until further preparation. Arthropod densities were estimated in summer 2007 by sampling 10 plots (d = 0.7 m^2^) per island using the converted leaf blower. For more detailed descriptions of the sampling method for terrestrial arthropod densities see Kolb et al. 2010 [7].To estimate soil nutrients, we collected three soil samples on 10 islands, with a soil core (d = 3 cm) at 0–10 cm depth. Samples were divided into two layers 0–5 cm and 5–10 cm depth, cooled directly after sampling and stored frozen until sieving through a 2 mm mesh sieve.

In water, we randomly collected 6 *Fucus vesiculosus* fronds with their associated invertebrate fauna from two opposing island sides. Per site we sampled 3 *Fucus* at 0.5–5 meter distance from the shoreline and at 0.5 to 2 m depth. Samples were immediately transferred to plastic bags and stored in the freezer until sorting. In the laboratory, invertebrate grazers (crustaceans: *Idotea* spp. and *Jaera albifrons* (Isopoda) and *Gammarus* spp. (Amphipoda), gastropod: *Theodoxus fluviatilis*) and Chironomidae larvae (Diptera) were sorted for further analysis. Epiphytic algae were scraped of the fronds and analyzed separately. For NC-content analysis, 5 to 6 *Fucus* fronds and their associated epiphytic algae and major invertebrate groups were used per island, whereas for the P analysis only 2 fronds were used, one from each sampling side. For detailed descriptions of the sampling method for brackish water invertebrate biomass, see Kolb et al. 2010 [40].

All necessary permits were obtained from the county administrative board in Stockholm. Sampling on islands which are not protected areas need no permission according to Swedish law. The field studies did not involve endangered or protected species.

### Carbon-nitrogen and Phosphorus Analyses

#### Soil

For P analyses in soil, samples with dominantly organic material were milled in a cychlotech mill to 0.5 mm, and sandy samples were homogenized in a machine mortar for maximum 3 minutes. Inorganic P was determined following extraction with 2% citric acid (1∶5 soil to extract solution) [41]. For ammonium and nitrate analysis, sieved soils were extracted with 2 M KCl (100 g soil per 250 ml liquid) for 2 hours for sandy samples and overnight for clayey soils. Soils were filtered and analyzed with flow injection analysis (Foss, Sweden), following the application notes AN 50/84 [42] and ASN 50-01/92 [43].

#### Primary producer and consumer

Before analysis, the plant material was dried at 55°C to constant weight and all invertebrates were freeze-dried. Phosphorus content (%P, dry mass basis) was assayed using persulphate digestion and ascorbate-molybdate colorimetry [44]. Nitrogen and carbon content (%N, %C, dry mass basis) was assayed in parallel to stable isotopes [7], [40] in an Isotope Ratio Mass Spectrometer type *Europa integra* or an elemental analyzer. In this analysis, samples were oxidized and reduced to CO_2_ and N_2_, respectively, which were measured with a thermal conductivity detector and IR-detection. Samples were prepared for P and CN analysis in one of three ways. Plants and algae were ground and subsamples were used for the analyses (P: 3–4 mg, CN: 1–3 mg). For arthropods smaller than 1.5 mg (P) and 0.5 mg (CN), we used pooled samples, while analyses of larger arthropods used whole individuals. Larger individuals of Lepidoptera, Isopoda and Coleoptera were lightly crushed and subsamples of 2–3 mg and 1–2 mg, respectively, were assayed for P and CN content.

### Statistics

We compared soil N (NH_4_^+^ and NO_3_^−^) between the three groups of cormorant islands and reference islands with linear mixed effects models, using island category as fixed effect and island and sample depth as random effect. With ANOVA and Tukey post-hoc tests we compared N:C, P:C, and N:P mass ratios of terrestrial plants among the four island categories. We tested for differences in N:C, P:C, and N:P mass ratios between trophic groups (herbivores, detritivores, predators, and chironomids) with linear mixed effects models, using trophic group as fixed effect and island category, island and taxonomic group as random effect. We also compared N:C, P:C, and N:P mass ratios of 1) the three major terrestrial trophic groups (herbivores, detritivores, and predators) and chironomids and 2) the taxonomic arthropod groups between active and abandoned cormorant islands and reference islands with linear mixed effect models using island category as fixed effect and either 1) island, order and family, or 2) island as random effects. Similarly, we compared N:C, P:C and N:P mass ratios of algae (*Fucus* and epiphytic algae) and brackish invertebrate groups among island categories using linear mixed effects models, with island category, adjusted wave exposure and their interaction as fixed effects and island as random effect. Best models were chosen with help of model comparison. In order to meet the assumption of normality and homoscedasticity, we adjusted wave exposure in all models ( = the difference between wave exposure of the sample side and mean wave exposure [wave exposure log-transformed]). All linear mixed effects models were done with the *nlme* package in R 2.12.1.

We examined the relationship between consumer and resource stoichiometry by the homeostasis coefficient *H*:
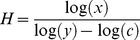
(1)where *x* is the resource elemental mass ratio (*N*:*C*, *P*:*C*, *N*:*P*), *y* is the consumer elemental ratio and *c* is a constant [27]. Equation 1 can be linearized as
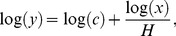
(2)suggesting that the degree of homeostasis can be found in a linear regression between the logarithm of a consumer elemental ratio and the logarithm of the resource elemental ratio. A given taxon is defined to be strictly homeostatic if its stoichiometry is tightly constrained across a wide variation in resource stoichiometry [27], [29]. The slope of the regression line (1/H) describes the strength of consumer homeostasis. In our study, we used this relationship not to test for elemental homeostasis of single species but of taxonomic groups. Deviation from strict homeostasis (1/H ≠ 0) can therefore indicate both changes in the species composition within the analyzed taxonomic group and elemental plasticity of single species. We conducted regression analyses for N:C, P:C, and N:P for 12 (7 for P:C and N:P) terrestrial taxonomic groups and 5 (3 for P:C and N:P) brackish invertebrate groups using terrestrial plants as resources for terrestrial herbivores and detritivores, chironomids as resources for spiders, and collembolans as resources for carabids. Carabids on the islands were Dyschirius spp. that are known to feed on collembolans. We regressed the elemental ratio (mean per island) of a given taxa on its resource (mean per island) across the sampled islands. Diet mixing models based on stable isotope analysis (δ15N and δ13C) in a former study indicated that all brackish grazers, except Idotea spp., mainly feed on epiphytic algae [40]. Hence we used epiphytic algae as a resource for brackish grazers and Fucus as a resource for Idotea spp.

We tested for effects of resource quality and quantity on terrestrial consumer densities with either multiple regressions or generalized linear models (glm) with a quasipoisson error structure depending on error distribution. Response variables were densities of arthropod groups, and explanatory variables were leaf NC-content, leaf PC-content and aboveground plant biomass (log-transformed). We chose the best model using the drop function in R and model comparison. To investigate if vegetation characteristics could also explain terrestrial arthropod densities we regressed densities against vegetation cover (sqrt-transformed) and plant species richness. Since these explanatory variable were correlated (t = 2.8, df = 15, p-value = 0.014, cor = 0.58) we tested their effects separately. We log-transformed arthropod densities if necessary in order to meet the assumption of normality and homoscedasticity. With linear mixed models we similarly investigated the relationship between resource quality and quantity and brackish invertebrate biomass (sqrt-transformed) using epiphytic algae N:C and P:C-ratios, epiphytic algae:*Fucus*-ratio (sqrt-transformed), adjusted wave exposure, and their interactions as fixed factors and island as random factor. Due to different sample sizes we analyzed N:C and P:C mass ratios separately. Best models were chosen through model comparison. Before analyses we checked for correlations between explanatory variables.

To investigate the relationship between consumer stoichiometry and consumer density/biomass response to cormorant nesting colonies we first calculated effect sizes among island categories for terrestrial (density) and brackish water (biomass) invertebrate groups. We compared the densities of the terrestrial taxonomic arthropod groups among three island categories (reference islands, abandoned and active cormorant islands) with ANOVA and Tukey post-hoc tests as described in Kolb et al. (2010) [7] (Table 1). We repeated the analysis with two island categories (reference and cormorant islands) (Table 1). Effect sizes for terrestrial arthropod densities (E1_terr_) were based on the ANOVA tables and defined as differences (mean ± SE) between reference islands and active cormorant islands (E1_terr_) and difference (mean ± SE) between reference islands and both active and abandoned cormorant islands (E2_terr_).

**Table 1:** ANOVA table for analysis of arthropod densities as a function of island category (reference island (RF), abandoned cormorant island (AB), active cormorant island (AC) and cormorant island (CO) including both abandoned and active islands).

Taxa	df,errordf	*F*	p	RF	AB	AC	E1	df,errordf	*F*	p	CO	E2
**Herbivore**												
Lepidoptera larvae	2, 16	**14.8**	**0.000**	5.9±1.9	18.2±6.13	32.0±4.02	1.82	1, 17	**26.7**	**0.000**	27.9±3.8	1.63
Auchenorrhyncha	2, 16	0.2	0.861	45.1±17.4	60.3±30.6	50.7±14.3	0.22	1, 17	0.2	0.653	53.5±12.6	0.32
Aphidina	2, 16	**4.8**	**0.023**	8.6±6.2	1.1±0.6	753±711	2.74	1, 17	**2.5**	**0.013**	528±499	1.68
Curculionidae	2, 16	**7.8**	**0.004**	8.1±2.4	35.4±7.0	3.3±1.4	−1.13	1, 17	0.2	0.685	12.9±5.3	−0.27
Chrysomelidae	2, 16	***3.0***	***0.081***	24.0±11.4	29.0±14.93	16.6±2.7	−1.2	1, 17	1.45	0.244	17.3±5.5	−0.71
**Detritivore**												
Isopoda	2, 16	2.1	0.155	203.7±80	52.6±48	139.2±48.9	−0.55	1, 17	1.9	0.188	113.2±38.0	−1.03
Collembola	2, 16	**6.2**	**0.010**	17.2±3.5	9.2±2.9	4.5±1.3	−1.44	1, 17	**8.9**	**0.009**	5.9±1.4	−1.16
Brachycerid diptera	2, 16	**13.9**	**0.000**	12±2.5	13.4±7.2	103.2±25.1	2.16	1, 17	**6.2**	**0.023**	76.3±22.1	1.41
**Chironomidae** *	3, 15	***3.2***	***0.052***	2.6±1.1	2.3±1.8	38.0±17.4*	1.54	3, 15				
**Predators**												
Araneidae	2, 16	1.9	0.183	7. 4±1.7	25.6±9.1	11.7±4.8	−0.22	1, 17	0.2	0.691	15.9±4.5	0.22
Linyphiidae	2, 16	1.1	0. 343	18.1±6.0	44.2±25.5	20.6 4.1	0.27	1, 17	1.3	0.273	28.7±8.3	0.45
*Tetragnatha* spp.	2, 16	**4.3**	**0.031**	1.0±0.7	6.8±1.8	2.3±0.9	1.59	1, 17	**5.9**	**0.026**	3.6±1.0	2.18
*Pachygnatha* spp.	2, 16	0.3	0.768	4.1±2.2	9.6±5.5	4.8±3.6	0.06	1, 17	0.1	0.734	6.3±2.9	0.41
Lycosidae	2, 16	**12.5**	**0.001**	19.8±4.5	22.8±5.9	02.8±1.6	−2.1	1, 17	**5.8**	**0.027**	8.8±3.6	−1.37
Parasitic hymenoptera	2, 16	**4.1**	**0.037**	4.8±0.9	6.9±3.2	10±1.0	0.90	1, 17	***4.4***	***0.052***	9.4±1.2	0.66
Coccinellidae	2, 16	**6.2**	**0.010**	2.9±0.6	3.7±1.5	12.3±0.42	1.43	1, 17	**7.1**	**0.016**	9.7±2.3	1.1
Carabidae	2, 16	***3.5***	***0.056***	1.54±0.32	2.44±0.65	0.73±2.7	−0.81	1, 17	0.3	0.578	6.0±2.3	−0.29
Staphylinidae	2, 16	0.9	0.416	4.1±1.4	7.3±2.0	5.8±2.0	0.45	1, 17	1.3	0.265	6.3±1.5	0.64
Nabidae	2, 16	0.7	0.524	1.3±0.5	4.7±4.2	5.1±2.9	0.68	1, 17	1.4	0.259	5.0±2.3	0.62
Formicidae	2, 16	0.4	0.670	71.3±31.1	88.2±46.1	50.9±21.6	−0.19	1, 17	0.0	0.976	62.1±19.8	−0.01

* For Chironomidae E2 = E1.

Shown are df, error df, F- and p- values, mean (±SE) individual numbers per island and effect size (E1 and E2). Significant (p>0.05) differences are bold in the table, marginal significant differences (p = 0.051–0.099) are cursive and bold.

Effect sizes for brackish invertebrates (E1_brack_) were calculated based on the difference in biomass (mean ± SE) between reference islands and the active cormorant islands with high nest density from linear mixed effect models in Kolb et al. (2010) [40] (Table S1). Effect sizes were weighted with 1/SE.

We tested for a relationship between consumer elemental mass ratios (N:C and P:C) and the effect size on consumer response with a regression analysis. Since soil and plant N-contents were only increased on active cormorant islands we regressed E1_terr_ and the consumer N:C mass ratios. Soil and plant P contents were increased on both abandoned and active cormorant islands, therefore we regressed E2_terr_ and the consumer P:C mass ratios. Finally, algal nutrient content (both N and P) was only increased around active cormorant islands with high nest densities and we regressed E1_brack_ with both consumer N:C and P:C mass ratios. We repeated the analysis with consumer nutrient limitation (L) as independent variable. Nutrient limitation (L) was defined as elemental mismatch between consumer and its resource on reference islands.
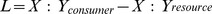



All statistical tests were performed in the free software R 2.10.0 or 2.12.1.

## Results

### Soil

**Figure 1:**
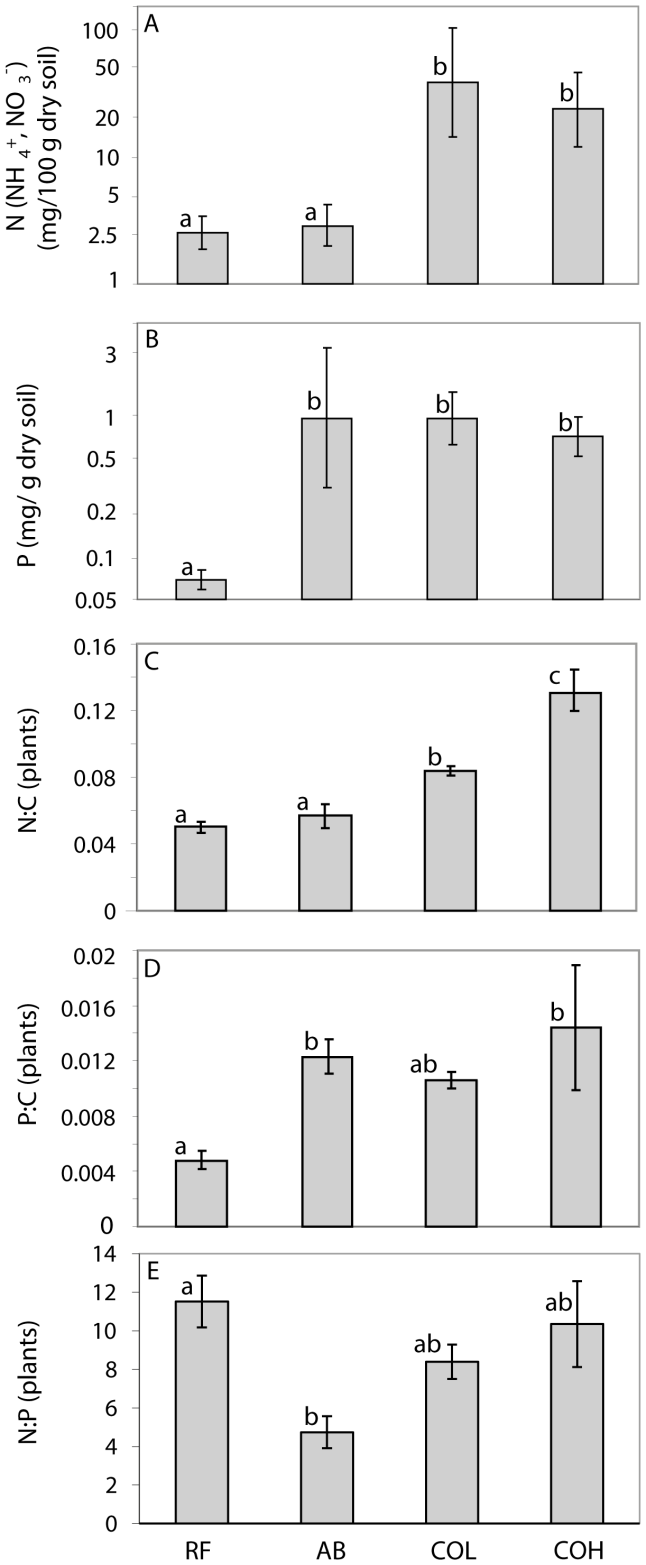
Soil (A) nitrogen (NH_4_^+^ and NO_3_^−^) (mg/100 g dry soil) and (B) phosphorus (mg/kg dry soil) content and (C–E) elemental ratios (mean ± SE) of herbs and grasses on reference islands (RF) (non-cormorant islands), abandoned cormorant islands (AB) and active cormorant islands with low (COL) and high (COH) nest density. Different letters indicate significant differences in linear mixed effect model (A and B) and post-hoc test (C–E).

Plant available N (NH_4_^+^ and NO_3_^−^) (mg/100 g dry soil) was 15-fold higher on islands with low cormorant nest density and 9-fold higher on islands with high nest density than on reference islands and about equal on abandoned and reference islands (F = 7.2, p = 0.021, dendf = 6, n = 50; Fig. 1A). Soil P content (mg/g dry soil) was higher on all cormorant islands, both abandoned (10-fold) and active (14-fold), than on reference islands (F = 8.5, p = 0.014, dendf = 6, n = 55; Fig. 1B).

### Autotrophs

**Figure 2:**
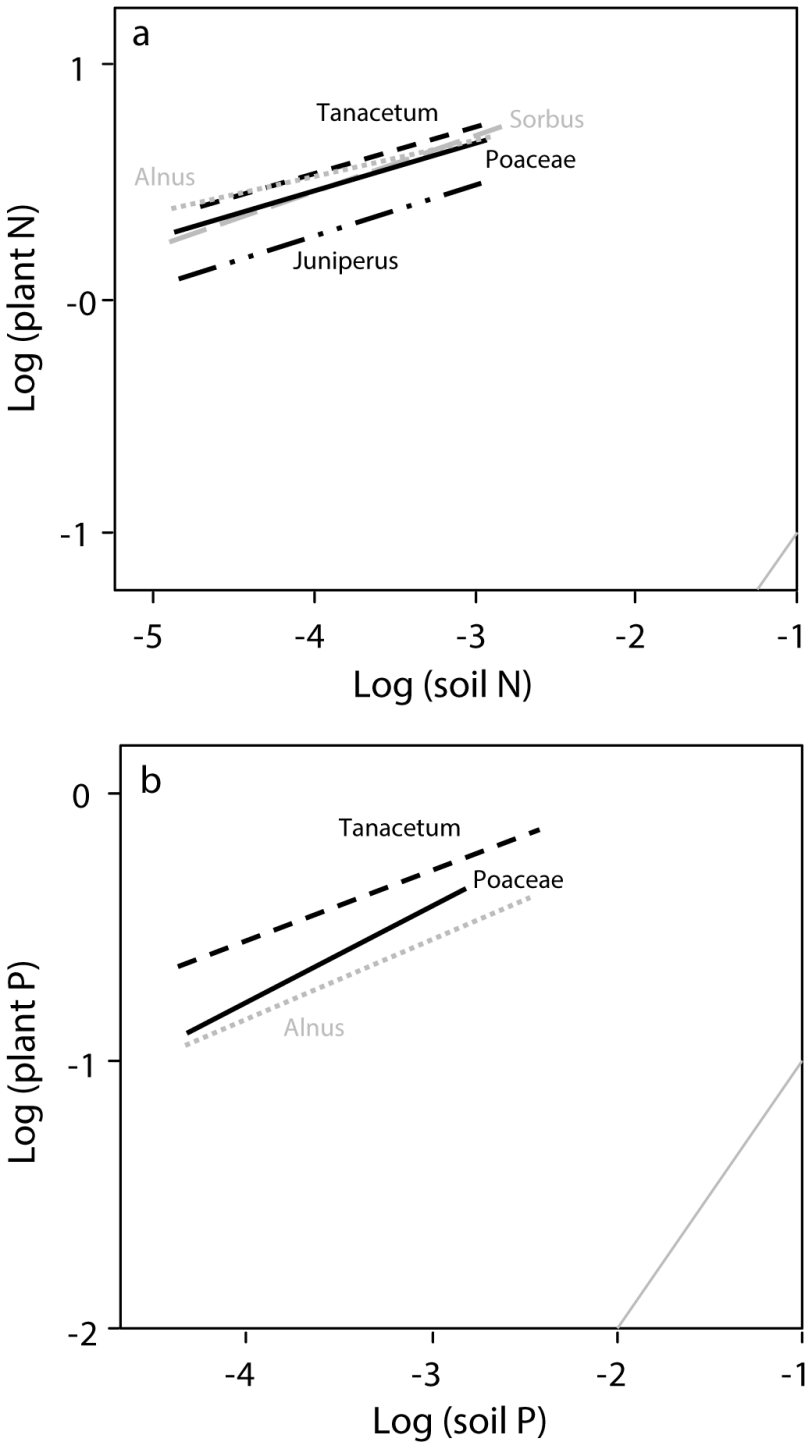
Relationship between soil and plant nitrogen (%N) (A) and phosphorus (%P) (B) on active, abandoned and reference islands. The horizontal range corresponds to the range of the soil N and P. The grey diagonal line represents the 1∶1 relation. See Table S3 for ANOVA tables.

Terrestrial plants generally had higher N:C and P:C mass ratios on active cormorant islands than on reference islands (Fig. 1C and D, Table S2). On abandoned nesting islands, plant N:C mass ratios were about equal to reference islands, while P:C mass ratios were enriched. Consequently, the plant N:P mass ratios on abandoned islands were lower than on reference islands (Fig. 1E). Taxonomic groups differed slightly in the response magnitude (Appendix 1). Herbs and grasses had 2.6-fold and 1.7-fold higher N:C mass ratios on islands with high and low nest density respectively than on reference islands. The P:C mass ratio of herbs and grasses was 3.0-fold higher on islands with high nest density, tended to be 2.2-fold higher on islands with low nest density and was 2.4-fold higher on abandoned cormorant islands than on reference islands. The N:P mass ratio of herbs and grasses was 2.4-fold lower on abandoned than on reference islands (Fig. 1C, D and E, Table S2). All terrestrial plant taxa deviated from a strict elemental homeostasis; their 1/H_%N_ varied between 0.15 and 0.23 and their 1/H_%P_ varied between 0.23 and 0.36 (Fig. 2, Table S3).

**Figure 3:**
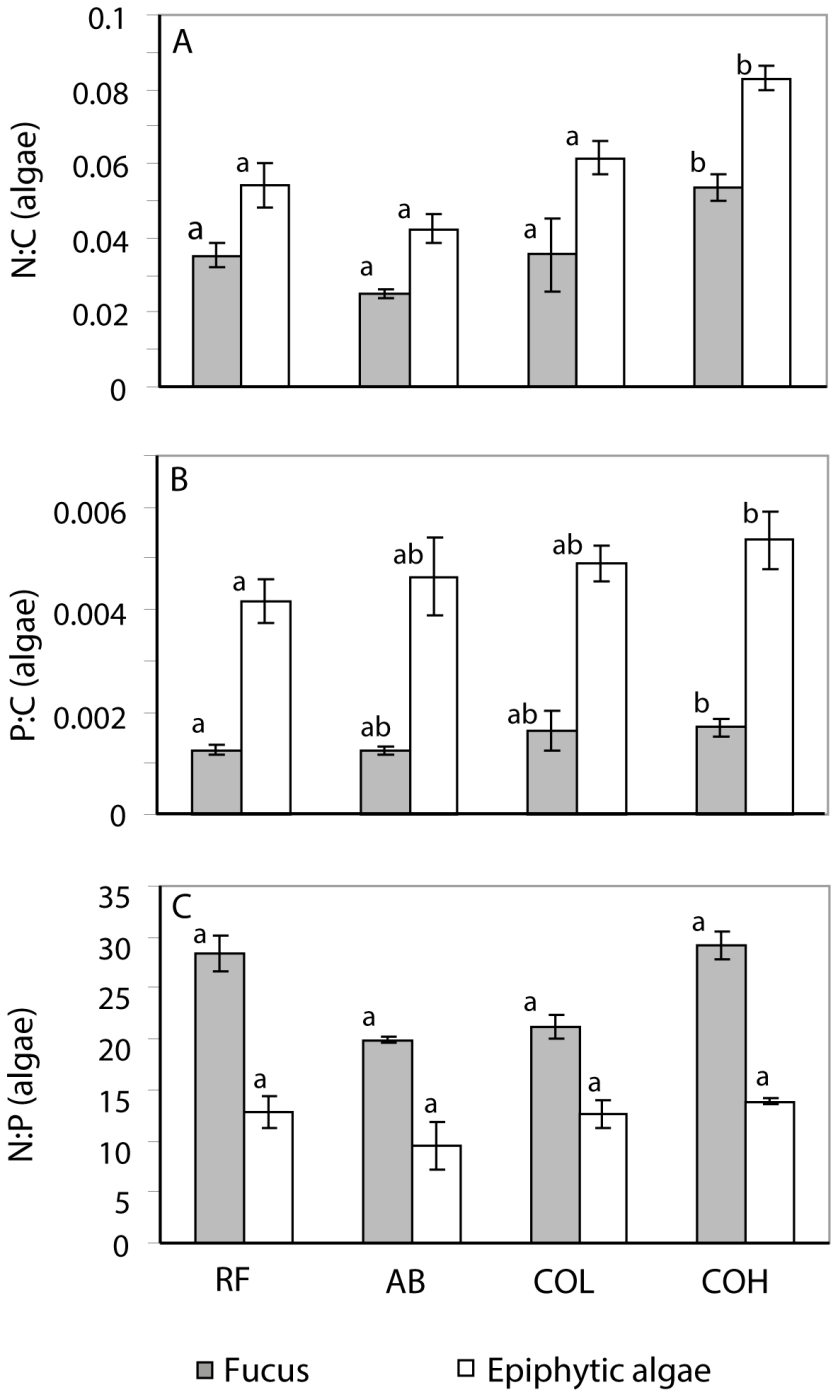
Elemental ratios (mean ± SE) of algae nearby reference (RF), abandoned (AB) cormorant and active cormorant islands with low (COL) and high (COH) nest density. Different letters indicate significant differences in linear mixed effect model.

Algae, generally, had smaller differences in elemental composition between island categories than terrestrial plants (Fig. 3, Table S4). Algae nearby cormorant islands with high nest density had higher N:C and P:C mass ratios than algae nearby reference islands, while algae from islands with low nest density and abandoned colonies had about equal N:C and P:C mass ratios as reference islands. The algal N:P mass ratios were about equal on active cormorant islands and reference islands. Furthermore, epiphytic algae N:C mass ratios decreased with increasing wave exposure (Table S4).

### Heterotrophs

The analysis of elemental mass ratios for terrestrial and brackish invertebrates revealed a wide variation among taxa and only small variations, for a few taxa, between island categories.

#### N:C

**Figure 4:**
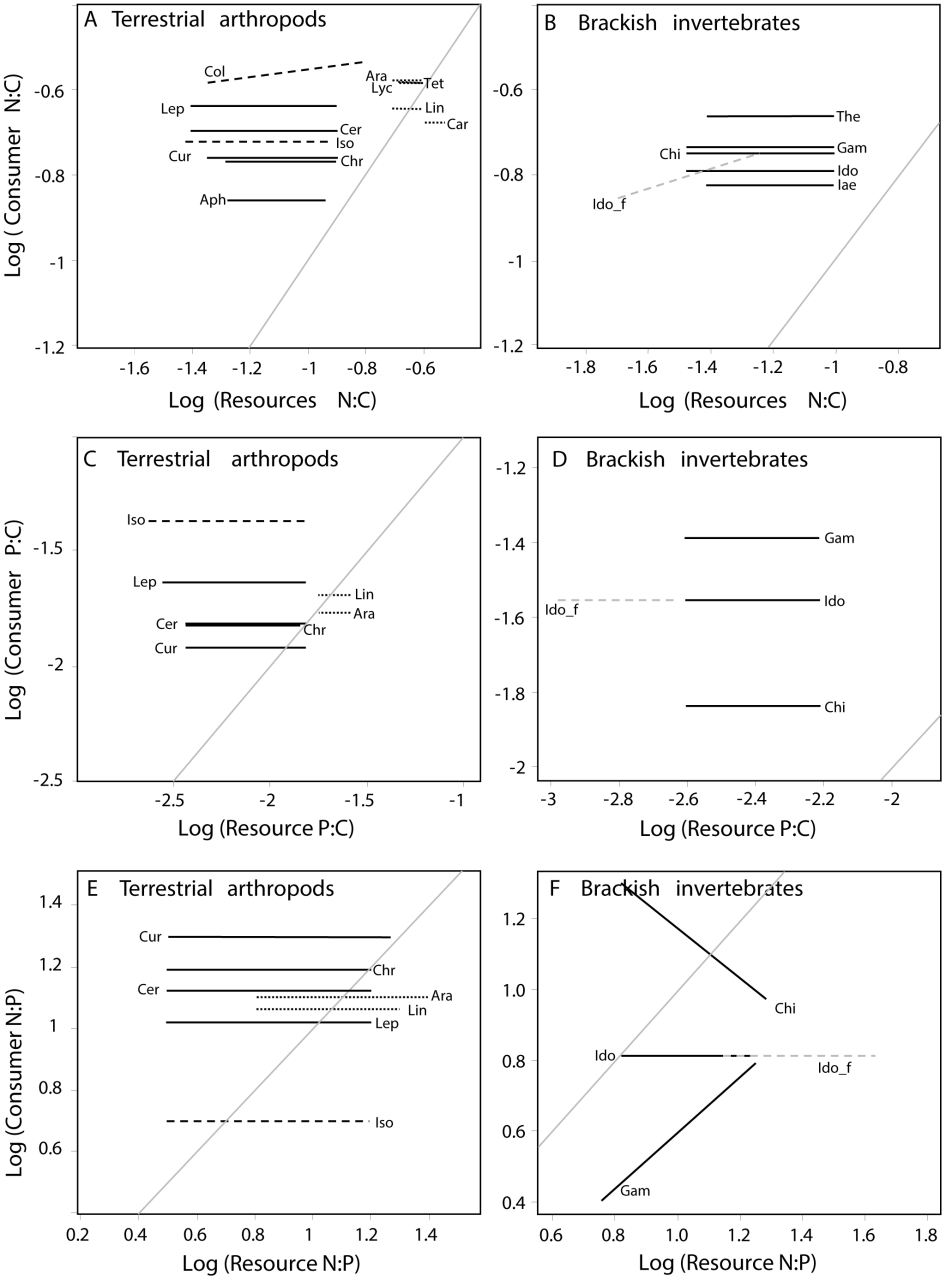
Relationship between consumer and resource N:C (A and B), P:C (C and D), and N:P (E and F) for terrestrial arthropods (A, C, E) and brackish invertebrates (C, D, F). Solid lines indicate herbivores, dashed lines detritivores, and dotted lines predators. The horizontal range corresponds to the data range of the resource. The grey diagonal line represents the 1∶1 relation. Resource: Plants: Col: Collembola, Iso: Isopoda, Lep: Lepidoptera larvae, Cer: Cercopidea, Cur: Curculionidae, Chr: Chrysomelidae, Aph: Aphids; Resource: adult Chironomidae: Ara: Araneidae, Lin: Linyphiidae, Tet: Tetragnathidae, Lyc: Lycosidae; Resource: Collembola: Car: Carabidae; Resource: Epiphytes: The: *Theodoxus fluviatilis,* Chi: Chironomidae larvae, Id: *Idotea* spp, Gam: *Gammarus* spp, Ia: *Jaera albifrons;* Resource*: Fucus:* Id_f: *Idotea* spp.

**Figure 5:**
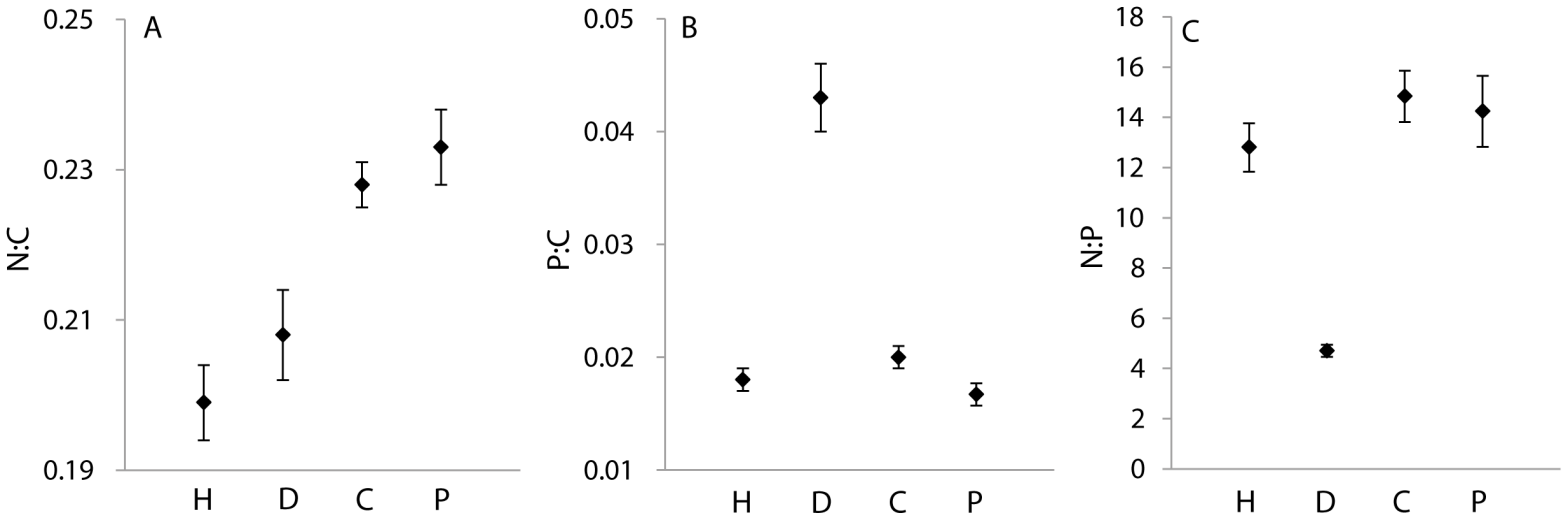
Mean (±SE) body N:C (A), P:C (B), and N:P (C) mass ratios for terrestrial herbivores (H), terrestrial detritivores (D), adult chironomids (C) and terrestrial predators (P).

The N:C mass ratios showed a 2-fold difference among terrestrial arthropods, collembolans had the highest and aphids the lowest N:C mass ratio (Fig. 4A). In general, terrestrial herbivores and detritivores consumed diets with lower N:C than themselves; that is, regression lines for these groups with their respective diet fell above the 1∶1 line. Herbivores on reference islands had a 4-fold higher N:C mass ratio than plants while this mismatch was only half on active cormorant islands. The three main terrestrial trophic groups (predators, herbivores and detritivores) and chironomids differed in their N:C mass ratios (F = 22.3, p<0.0001, den df = 540, n = 792) (Fig. 5A). Predators had generally higher N:C mass ratios than herbivores and detritivores but about equal N:C mass ratios to adult chironomids (Fig. 5A), but carabids feeding on collembolans were an exception. Collembolans had a higher N:C mass ratio than carabids, and their regression line fell under the 1∶1 line (Fig. 4A). Spiders feeding on adult chironomids had a N:C mass ratio quite similar to their potential prey (Fig. 4A). Brackish invertebrates consumed diets with lower N:C than themselves; this was especially pronounced for *Idotea* feeding on *Fucus* (Fig. 4B).

The N:C mass ratios of all terrestrial trophic groups were about equal among island categories (Table 2, Table S5), and differed among categories only for two (Curculionidae and Linyphiidae) of 20 arthropod groups (Table 2). This difference was lost after Bonferroni correction. Accordingly, strict N:C homeostasis was observed for 10 of 11 taxonomic groups (Fig. 4A, Table S6). However, the deviation of collembolans from strict homeostasis lost its significance after Bonferroni correction.

**Table 2:** Results of linear mixed effects model (lme) testing for differences in elemental ratios of terrestrial arthropods and insects between reference islands and cormorant islands (abandoned and active).

Taxa	N:C				P:C				N:P			
	n	is	*F*	*p*	n	is	*F*	*p*	N	is	*F*	*p*
**Herbivores**	141	15	0.3	0.78	53	16	1.2	0.34	53	16	0.2	0.83
Aphidina	7	5	2.3	0.31								
Cercopidea	45	14	1.0	0.39	**13**	**13**	**4.4**	**0.04**	13	13	3.4	0.08
Lepidoptera larvae	41	15	0.4	0.74	15	15	1.0	0.39	15	15	1.0	0.39
Chrysomelidae	25	16	0.1	0.89	14	14	0.0	0.97	14	14	1.2	0.33
Curculionidae	**26**	**16**	**4.3**	**0.04**	11	11	1.7	0.24	11	11	0.4	0.69
**Detritivore**	84	15	0.1	0.88	14	14	0.9	0.45	14	14	1.2	0.36
Isopoda	70	15	0.3	0.72	14	14	0.9	0.45	14	14	1.2	0.36
Collembola	14	10	4.4	0.06								
Brachycerid diptera	32	11	13	0.32								
**Chironomidae**	51	15	3.6	0.06	13	13	1.3	0.31	13	13	0.1	0.92
**Predators**	445	20	1.4	0.28	40	14	0.4	0.69	41	15	0.1	0.88
Araneidae	65	16	0.4	0.69	13	13	0.3	0.75	13	13	0.2	0.86
Linyphiidae	**39**	**14**	**5.6**	**0.02**	10	10	0.2	0.81	10	10	1.4	0.31
Tetragnathidae	57	11	0.0	1.0								
Lycosidae	55	11	4.3	0.05								
Coccinellidae	34	14	1.0	0.41	12	12	0.0	0.97	12	12	0.4	0.68
Carabidae	53	16	1.3	0.31	6	6	0.2	0.81	6	6	0.3	0.73
Staphylinidae	23	9	1.7	0.27								
*Nabis* spp. (Nabidae)	36	9	0.3	0.74								
Formicidae	58	13	1.0	0.40								

Shown are the number of samples (n), number of islands (islands), F- and p- values from ANOVA for lme. Significant (p>0.05) differences are bold in the table.

The N:C mass ratios of brackish invertebrate differ only slightly for *Jaera albifrons* between active cormorant islands with high nest density and reference islands (Table 3). *Jaera albifrons*, furthermore, showed a significant interaction between island category and wave exposure. N:C of chironomids decreased with wave exposure (Table 3). All five brackish invertebrates feeding on epiphytic algae were strictly homeostatic. *Idotea* spp. feeding on *Fucus* had a 1/H_N:C_ ≈ 0.25 (Fig. 4B, Table S6).

**Table:** **Table 3.** Results of linear mixed effects model (lme) testing for differences in elemental ratios (mean ± SE) of brackish invertebrates between reference islands and cormorant islands (abandoned and active cormorant islands with low and high nest density).

	n	is	F	p	Slope	Mean± SE
					(mean ± SE)	
**N:C**						
**Chironomidae**	91	17				
Island category (df = 3)			1.4	0.278		0.182±0.002
Wave exposure (df = 1)			7.0	0.010	−0.009±0.003	
Theodoxus fluviatilis	76	15				
Island category (df = 3)			1.2	0.368		0.221±0.003
***Gammarus*** ** spp**	121	17				
Island category (df = 3)			1.8	0.204		0.186±0.003
***Idotea*** ** spp**	142	17				
Island category (df = 3)			1.9	0.177		0.161±0.002
***Jaera albifrons***	66	15				
Island category (df = 3)			4.7	0.025		0.151±0.003
Wave exposure (df = 1)			0.1	0.708		
Island×Wave (df = 3)			17.8	<0.0001		
**P:C**						
**Chironomidae**	26	14				0.014±0.001
Island category (df = 3)			1.9	0.201		
***Gammarus*** ** spp**	34	17				
Island category (df = 3)			0.7	0.578		0.041±0.002
***Idotea*** ** spp**	30	15				
Island category (df = 3)			2.2	0.145		0.028±0.001
**N:P**						
**Chironomidae**	26	14				
Island category (df = 3)			2.6	0.112		13.67±0.83
***Gammarus*** ** spp**	34	17				
Island category (df = 3)			1.0	0.439		4.99±0.37
***Idotea*** ** spp**	30	15				
Island category (df = 3)		i	1.4	0.297		6.47±0.39

Shown are the number of samples (n), number of islands (islands), F- and p- values from ANOVA for lme, the slope for wave exposure, and mean ± SE over all islands. Significant effects in bold (p<0.05).

#### P:C

The P:C mass ratios among terrestrial arthropods showed a 4.4-fold difference; isopods had the highest and beetles had the lowest P:C (Fig. 4C). Herbivores on reference islands had a 3.3-fold higher P:C than plants while this mismatch was lower (1-fold) on active cormorant islands. Beetles on cormorant islands fed on plants with similar P:C as themselves. Other herbivores and detritivores had diets with lower P:C than themselves. Linear mixed equation models showed that P:C differed among trophic groups (F = 67.8, p<0.0001, den df = 34, n = 121), due to the extremely high P:C of isopods (Fig. 5B). Predators did not have higher P:C than herbivores, spiders had P:C mass ratios equal to or lower than their prey (chironomids). Brackish invertebrates showed a 2.8-fold variation in their P:C, furthermore, they were strongly enriched in P compared to their food (Fig. 4D).

The P:C mass ratios of all terrestrial and brackish trophic groups were about equal among island categories, and only two taxonomic groups (Cercopidae and *Idotea*) differed among categories (Table 2 and 3), but these significances were marginal and lost after correcting for multiple tests. All terrestrial and brackish invertebrate groups showed P:C homeostasis (Fig. 4 C and D, Table S6).

#### N:P

N:P mass ratios differed between detritivores (isopods) and the other trophic groups (F = 13.3, p<0.0001, den df = 102, n = 121) (Fig. 5C). Insects and spiders had higher N:P than isopods. Beetles and Cercopidae had higher to equal N:P than their resources. The regression lines of the other taxa crossed the 1∶1 line, thus feeding on diets with both lower and higher N:P than themselves (Fig. 4E). Brackish crustaceans were depleted in P compared to their diet whereas chironomids varied strongly relative to their diet (Fig. 4F). The N:P mass ratios of all terrestrial trophic and taxonomic groups were about equal among island categories (Table 2). Similarly, brackish invertebrates showed no variation in N:P among the island categories (Table 3). All terrestrial taxonomic arthropod groups had tightly regulated N:P homeostasis (Fig. 4E, Table S6). Of three brackish invertebrates taxa, one was strictly homeostatic (*Idotea*), one had a positive (*Gammarus*) and one had a negative (*Jaera albifrons*) regression slope (Fig. 4F, Table S6).

#### Density responses

Among invertebrate groups 17 out of 25 showed differences in density or biomass among island categories (Table 1, Table S1). Comparing reference islands with cormorant islands, both active and abandoned, two terrestrial arthropod groups had lower densities and four groups had higher densities on cormorant islands. Comparing only active cormorant islands and reference islands, two arthropod groups had lower density and five had higher densities on cormorant islands. Finally, comparing abandoned cormorant islands and references islands, three groups had higher densities on cormorant islands.

When testing plant quality or quantity effects we found that the densities of 6 taxonomic arthropod groups (Aphidina, brachycerid Diptera, Chironomidae, Coccinellidae, and parasitic Hymenoptera) were positively and 5 groups (Chrysomelidae, Curculionidae, Collembola, Carabidae, and Lycosidae) were negatively correlated with leaf NC-content (Table 4). The density of lepidopteran larvae and Curculionidae were positively, the density of collembolans negatively correlated with leaf P:C-content. Aboveground plant biomass was positively related to six taxonomic groups (Cercopidae, Chrysomelidae, Carabidae, Coccinellidae and Tetragnathidae) (Table 4). Out of the five brackish invertebrate groups only the biomass of Chironomidae was positively correlated with epiphytic algae:*Fucu*s-ratio (Table 5). The biomass of *Theodoxus fluviatilis* showed a positive relationship to algal P:C-content (Table 5). Algal N:C-content did not have an effect on invertebrate biomass. Wave exposure affected the biomass of *Theodoxus, Gammarus, Idotea,* and *Jaera albifrons* negatively (Table 5). Wave-exposure and epiphytic algae:*Fucus-*ratio had a positive interactive effect on the biomass of *Theodoxus fluviatilis* and *Idotea* (Table 5).

**Table 4:** Results of linear regressions (lm) and generalized linear models (glm) testing for a linear relationship between plant quality (leaf N:C and P:C-content) and plant quantity (aboveground plant biomass (g/62.5 cm^2^) and arthropod densities.

Taxa	mo	Leaf NC-content	Leaf PC-content	Plant biomass	
**Herbivores**					
Aphidina	lm	(+)			F = 7.4, p = 0.017,
					df = 14, R2 = 29.8%
Cercopidea	glm			(+)	P(χ2) 0.015
					df = 14
Lepidoptera larvae	lm		(+)		F = 33.0, p<0.0001
					df = 13, R2 = 69.6%
Chrysomelidae	lm	(−)		(+)	
		t = −6.3, p<0.0001		t = 4.6, p<0.001	
Curculionidae	glm	(−)	(+)		
		t = −3.5, p = 0.004,	t = 3.1, p = 0.008		
		P(χ2) = 0.001, df = 14	P(χ2) 0.002, df = 13		
Herbivorous Heteroptera	lm				
					
**Detritivore**	lm				
Isopoda	lm				
					
Collembola	lm	(−)	(−)		F = 16.1, p<0.001,
		t = −3.6, p<0.003	t = −2.5, p<0.026		df = 13, R2 = 66.8%
Brachycerid	lm	(+)			F = 28.8, p<0.001,
Diptera					df = 14, R2 = 62.3%
**Chironomidae**	lm	(+)			F = 10.0, p<0.006,
					df = 14, R2 = 37.6%
**Predators**					
Araneidae	lm				
Linyphiidae	lm				
Tetragnathidae	glm			(+)	
				t = 2.7,p = 0.016,	
				P(χ2) <0.002, df = 14	
Pachygnatha	lm				
Lycosidae	glm	(−)			
		t = −2.8,p = 0.015,			
		P(χ2) <0.002, df = 14			
Coccinellidae	glm	(+)		(+)	
		t = 6.2, p<0.0001		t = 4.7, p<0.001	
		P(χ2) <0.0001, df = 14		P(χ2) <0.0001, df = 13	
Carabidae	lm	(−)		(+)	F = 14.2, p<0.001,
		t = −3.9, p = 0.002		t = 3.3, p = 0.005	df = 13, R2 = 63.8%
Staphylinidae	lm				
Nabis spp.	glm				
Formicidae	lm				
Parasitic	lm	(+)			F = 18.5, p<0.001,
hymenoptera					df = 14, R2 = 53.8%

Shown are the direction of effect positive (+) and negative (−). Only significant results are shown (p>0.05).

**Table 5:** Results of linear mixed effect models (lme) testing the relationship between algal N:C (P:C)-content, epiphytic alage:*Fucus*-ratio (Epi:Fu-ratio) (sqrt-transformed), and wave exposure to brackish invertebrate biomass (mg dry-weight invertebrate/g dry-weight algae) (sqrt-transformed).

	n	is	*F*	*p*	Slope
					(mean ± SE)
**Chironomidae**					
Epi:Fu-ratio	99	17	17.1	0.0001	0.86±0.21
Theodoxus fluviatilis					
Epi:Fu-ratio	99	17	0.8	0.361	0.83±0.90
Wave exposure	99	17	23.3	<0.0001	−1.82±0.38
Epi:Fu-ratio * Wave exposure	99	17	8.3	0.005	2.25±0.78
Algal P:C-content	34	16	7.9	0.013	545±194
***Gammaru*** **s spp**					
Wave exposure	99	17	4.4	0.040	−0.67±0.32
***Idotea*** ** spp**					
Epi:Fu-ratio	99	17	0.2	0.640	0.17±0.41
Wave exposure	99	17	5.7	0.019	−0.35±0.14
Epi:Fu-ratio * Wave exposure	99	17	6.6	0.012	0.91±0.35
***Jaera albifrons***					
Wave exposure	99	17	18.0	0.0001	−0.15±0.04

Shown are the number of samples (n), number of islands (islands), F- and p- values from ANOVA for lme. Significant effects in bold (p<0.05). Algal N:C-content did not have any significant effect and is therefore not shown in the table.

**Figure 6:**
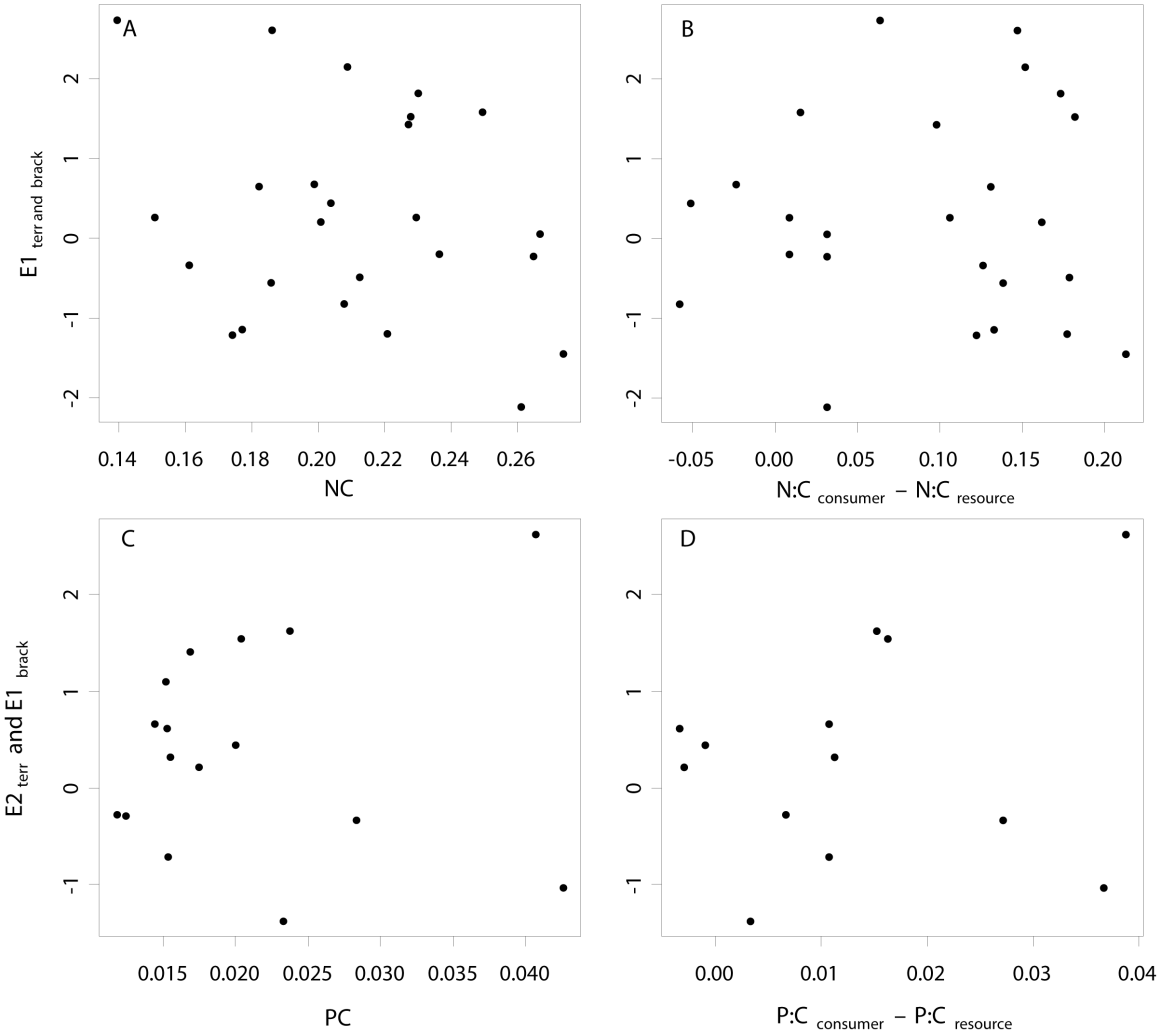
Relationship between the elemental mass ratios (N:C and P:C) of invertebrate taxa(A and C), the elemental mismatch between consumers and their resources (B and D) and the effect size of terrestrial arthropods (density) between reference and active cormorant islands (E1_terr_) and reference and cormorant islands (E2_terr_) and of brackish water invertebrates (biomass) between reference and active cormorant islands with high nest density (E1_brack_).

Among other vegetation variables, vegetation cover correlated with the density of the same arthropod groups as leaf N:C-ratios (expect Coccinellidae), but inversely. Plant species richness correlated positively with the density of Collembola and Lycosidae and negatively with the density of brachycerid Diptera and Chironomidae (Table S7). Aboveground plant biomass was not correlated with leaf N:C or P:C content, vegetation cover or plant species number. Plant N:C and P:C-content were not correlated. Vegetation cover correlated strongly negative with leaf N:C-content (t = −4.4, p<0.001, df = 15) and tended to correlate negatively with leaf P:C-content (t = −2.0, p = 0.060, df = 14). Plant species richness tended to correlate with leaf P:C-content (t = −1.9, p-value = 0.080, df = 13). The estimated effect sizes for consumer density responses did not correlate with either consumer elemental ratios (N:C: F = 0.7, p = 0.42, df = 23; P:C: F = 0.1, p = 0.72, df = 14) or consumer nutrient limitation (L_NC_: F = 0.0, p = 0.87, df = 23; L_PC_: F = 0.6, p = 0.47, df = 11) (Fig. 6).

## Discussion

We found that nesting cormorants radically changed soil nutrient composition, plant nutrient content and assumingly resource quality to herbivores and detritivores. In contrast, invertebrates generally had only small differences in either N:C or P:C mass ratios between island categories. On reference islands, there was a large difference in N and P content between plants and plant consumers (terrestrial and aquatic herbivores and detritivores) similar to previous studies [14], [45], [46], potentially leading to nutrient limitation [8], [12], [14], [17], [46]. Due to the higher plant nutrient content on active cormorant islands, differences in N and P content were much smaller between plants and plant consumers on these islands. We should therefore according to theories on ecological stoichiometry expect herbivore populations to be less nutrient limited on cormorant than on reference islands. Generally, nutrient limitation is assumed to be very common among terrestrial herbivores and detritivores, with more evidence for N than P limitation in the literature. Terrestrial systems are often assumed to be N limited and arthropod densities frequently increase with an increased plant N [8], [17], [47]–[49]. Although recent stoichiometry data suggests that for insect herbivores P limitation may be as severe as N limitation [45], effects on terrestrial arthropod fitness or population growth are relatively unexplored [50], [51]. Our study indicates that food for most herbivorous insects is more deficient in N than P (Fig. 4E). This relative N limitation seems even more severe on abandoned cormorant islands since plants on these islands show extremely low N:P ratios. Furthermore, crustaceans have much lower N:P mass ratios than insects and most likely have a higher P demand (Fig. 4E and F). Due to the small variation in invertebrate stoichiometry, food quality for predators and other higher order consumers shows small differences between island categories, suggesting no differences in nutrient limitation between reference and cormorant islands. Nutrient limitation also seems less common and severe among predators than among herbivores and detritivores since many predators have elemental ratios similar to their prey.

The differences in soil and plant nutrient content among island categories and between land and water suggest that the fate of N and P in cormorant guano depend on cormorant density and colonization history. As expected, islands with active nesting by cormorants generally had much higher soil N and P contents than reference islands, and plant N content increased with an increasing cormorant density. On abandoned islands, however, soil N content was similar to reference islands whereas soil P content was similar to active nesting islands, and this change was also reflected in the P:C and N:C mass ratios of plants. At the same time, plant biomass tended to be higher on abandoned than on reference islands [7], suggesting that the N input promoted plant growth and incorporation in plant tissue. It is also likely that the difference in N and P response is due to that P is retained better in soils than N [52]. On active cormorant islands with high nest density, N and P leached from the islands into surrounding waters and caused increased N and P content of brown and green algae. On islands with low nest densities, plants were better able to absorb the added nutrients. This difference is perhaps not surprising as islands with low nest density generally had high aboveground plant biomass and more or less continuous plant cover whereas islands with high nest density had very patchy plant cover with much bare ground due to the toxic effect from high amounts of guano on plants [7]. Furthermore, plant species richness was lower on active cormorant islands than on reference islands [5]. These differences in island vegetation may also explain differences in arthropod densities between island categories [5].

In contrast to plant nutrient content, invertebrates had only small differences in N:C or P:C mass ratios between island categories. To estimate these ratios, we pooled samples of related species and the small differences can therefore not be directly interpreted as elemental homeostasis. The mean nutrient content in invertebrates could also be affected by changes in species composition among islands with and without cormorant nesting colonies. Kolb et al. (2012) [5] showed that the species compositions of coleopterans and spiders were different on islands with and without cormorants. It might be that less homeostatic species suffer higher mortality in which case assemblage homeostasis would not indicate homeostasis of all members. Due to this change in species composition, we are currently unable to identify the reason for the apparent homeostasis of invertebrates in this study. However, it seems that the most likely hypothesis is still that the invertebrates are truly homeostatic. Our argument is that if the invertebrate species were not homeostatic then, most likely, their N and P content would be expected to be higher on islands with more available N and P [9], [29]. If this is true, we could only observe the apparent homeostasis if there is a shift towards species with a lower N and P content on islands with nesting cormorants. While we cannot exclude this possibility, it seems like a less likely hypothesis since nutrient rich species are more favored by high resource nutrient content than nutrient poor species [27], [35], [53].

Irrespective of the cause for the apparent homeostasis of invertebrate herbivores and detritivores, we can conclude that for the next trophic level, predators or parasitoids, there is no obvious change in diet quality between island categories in this study. Thus, we found no evidence for the hypothesis that cascading bottom-up effects may be mediated by qualitative changes in primary consumers [23], [26], [54]. In our parallel study, we observed increased densities of coccinelids and parasitic hymenopterans on active cormorant islands but this observation is more likely explained by increased prey (aphids) and host (lepidopterans and dipterans) densities [5].

A previous study in Neotropical streams found, in contrast to our study, that most taxonomic invertebrates groups in chronic P enriched Neotropical streams had two-fold higher P content than invertebrates in low-P streams [28]. The authors explained this P enrichment with deviation of strict homeostasis at the species level [28]. There might be several reasons for the discrepancy between our study and the study by Small and Pringle. First, their study was performed in an area where the P enrichment of the river was caused by strong long-term (over millennia) input of solute-rich groundwater while the islands in our study system have been colonized by cormorants for at most 16 years. Second, the P enrichment in the basal resource of the Neotropical streams is much higher than on or around the cormorant islands and it is possible that only strong enrichment in basal resources cause primary consumers to deviate from strict homeostasis. Third, aquatic invertebrates might be more prone to deviation from strict homeostasis than terrestrial arthropods. Fourth, since sample sizes in some groups were rather small it might be that we were unable to detect deviation from strict homeostasis due to a lack of power or especially variable data.

Using data from two parallel studies [7], [40], we relate the stoichiometry of invertebrate taxa, and eventual mismatches between consumer and resource stoichiometry, to observed density differences between islands with and without nesting cormorants. The chemical analyses indicated a two- to four-fold difference in the N:C and P:C mass ratios among taxa and an even larger potential trophic mismatch. Despite this range of elemental ratios, we found no relationship between invertebrate stoichiometry and density differences among island categories. It thus seems that consumer stoichiometry is not a useful predictor of arthropod responses to variation in resource nutrient content. We note that theories on ecological stoichiometry like the growth rate theory, which relates body P content to the RNA content and indirectly to individual growth rates [35], focused originally on aquatic species under P-saturated conditions and the growth rate theory has mainly been applied in aquatic systems, notably on Daphnia [27], [37], [55]–[57], but see [10].

When further investigating effects of plant quality (leaf/algae NC and PC-content) and quantity (aboveground plant biomass/epiphytic algae:*Fucus*-ratio) on consumer density/biomass we did not find a common positive relationship between resource quality and consumer density. Aphids were the only herbivores which showed a positive relationship to leaf N:C-content and lepidopteran larvae and weevils were the only two terrestrial arthropod group which showed a strong positive relationship to leaf P:C-content. Lepidoptera were also the group together with isopods with the highest P:C ratio (Fig. 4C and E) and thus potentially have the highest P demand. There was also a positive density response of *Theodoxus fluviatilis* to algal P:C-content, despite that this taxon has a fairly low P:C ratio, much lower than isopods which did not show any density response [58].

Several taxonomic groups had surprisingly a negative density response to leaf N:C-content. The literature contains few examples where arthropod densities or fitness decrease at a high resource nitrogen content [59]. Experiments on aquatic snails, however, show that both very high and very low P:C ratios in the microbial resource are detrimental for the performance of grazing snails [60]. To explain this pattern, Elser et al. (2005) [60] suggested that organisms in low nutrient environments might be selected towards reduced P-demand and are thus unable increase growth at very high P:C ratios in their resources. These organisms are likely to have an efficient P assimilation which may lead to P-poisoning when resource P:C ratios are high. This explanation seem less likely for at least on group, Collembola, having a negative relationship both leaf P:C and N:C content since they showed the highest N:C mass ratio of all taxonomic groups investigated (Fig. 4A).

The limited evidence that N and P content was a prime determinant of invertebrate density or biomass in our system does not imply that arthropods in our system are not nutrient limited but rather that other factors are more important determinants of population growth. The connection between N and P contents and population growth rates may be weak because other factors are more important determinants of population growth. For instance, P contents among terrestrial invertebrate species seem to be best predicted by variation in body size [50], [61] and not by variation in diet, and there may be other reasons why small species often have a high growth rate. Several abiotic and biotic factors are certainly different between islands with and without cormorants, such as vegetation cover, plant species richness, and composition, habitat complexity and resource abundance [5], [7]. Our analysis suggests that several herbivores groups (Cercopidea and Chrysomelidae) rather benefit from increased plant biomass. Similarly, an increased epiphytic-*algae*:Fucus ratio affect chironomid larvae biomasses positively. Furthermore, since leaf N:C-content and vegetation cover were strongly negative correlation it is unclear which variable was most important in determining arthropod densities.

To conclude, our study indicates that ecological stoichiometry seem less able to predict arthropod responses to variation along a resource gradient. It is unclear whether this limited predictability is due to that other factors than nutrient content are more important in our complex system, or that the conditions for understanding nutrient limitation is different in terrestrial arthropods than planktonic crustaceans.

## Supporting Information

Table S1 **Results of linear mixed effect models testing for differences in biomass (mg dry-weight invertebrates per g dry-weight algae) (square root transformed) of algae and aquatic invertebrate groups between reference (RF) (i.e. non-cormorant) islands and 3 categories of cormorant islands (abandoned [AB], active with low [COL] and high [COH] nest densities).** Samples were collected in the surrounding water bodies of 17 islands in the Stockholm archipelago, Baltic Sea.
(DOCX)

Table S2 **ANOVA table of analysis of plant elemental ratios as function of island category.** Shown are mean ± SE for the four island categories (reference islands (RF), abandoned cormorant island (AB), active cormorant island with low (COL) and high (COH) nest density.
(DOCX)

Table S3 **Statistic summary for regressions between soil and plant %N and %P.**
(DOCX)

Table S4 **Results of linear mixed effects model (lme) testing for differences in elemental ratios (mean ± SE) of brackish invertebrates between reference islands (RF) and cormorant islands (abandoned (AB) and active cormorant islands with low and (COL) high (COH) nest density).** Shown are the number of samples (n), number of islands (islands), F- and p- values from ANOVA for lme, the slope for wave exposure, and mean ± SE for the four island categories.* indicate significant difference (p<0.05), ^•^ marginal significant difference (p<0.1) from reference islands.
(DOCX)

Table S5 **Results of linear mixed effects model (lme) testing for differences in elemental ratios (mean ± SE) of terrestrial arthropods and insects between reference islands (RF) and cormorant islands (abandoned (AB) and active (AC)).** * indicate significant difference (in bold) (p<0.05) from reference islands.
(DOCX)

Table S6 **Summary statistic for regressions between resources and consumer N:C, P:C, and N:P.** Slopes provided are adjusted slopes, that is adjusted slopes are equal to the calculated slopes if regressions were significant (in bold); slopes from insignificant regressions (p>0.05) were set to zero.
(DOCX)

Table S7 **Results of linear regressions (lm) and generalized linear models (glm) testing for a linear relationship between plant quality (leaf N:C and P:C-content) and plant quantity (aboveground plant biomass (g/62.5 cm2) and arthropod densities.** Shown are the direction of effect positive (+) and negative (−). Shown are only significant results (p<0.05).
(DOCX)
