# Supplementary material for: Ecological Stoichiometry and Density Responses of Plant-Arthropod Communities on Cormorant Nesting Islands
Source: PLoS One. 2013 Apr 23;8(4):e61772. doi: 10.1371/journal.pone.0061772 (PMC3634001; doi:10.1371/journal.pone.0061772)
Supplement: Table S1 — Results of linear mixed effect models testing for differences in biomass (mg dry-weight invertebrates per g dry-weight algae) (square root transformed) of algae and aquatic invertebrate groups between reference (RF) (i.e. non-cormorant) islands and 3 categories of cormorant islands (abandoned [AB], active with low [COL] and high [COH] nest densities). Samples were collected in the surrounding water bodies of 17 islands in the Stockholm archipelago, Baltic Sea. (DOCX) [file pone.0061772.s001.docx]

**Table S1**

| Taxa |  |  | Island category | Wave exposure | Island category × wave exposure |
| --- | --- | --- | --- | --- | --- |
| Chironomidae | den df | 81 | 13 |  |  |
|  | F |  | 2.5 |  |  |
|  | p |  | 0.057 |  |  |
|  | slope (mean ± SE) |  |  |  |  |
| *Theodoxus fluviatilis* | den df | 84 | 13 | 84 |  |
|  | F |  | 2.7 | 21.9 |  |
|  | p |  | 0.089 | <0.0001 |  |
|  | slope (mean ± SE) |  |  | –1.2 ± 0.3 |  |
| *Gammarus* spp | den df | 81 | 13 | 81 | 81 |
|  | F |  | 7.6 | 0.6 | 6.4 |
|  | p |  | 0.004 | 0.458 | 0.001 |
|  | slope (mean ± SE) |  |  | RF: –0.2 ± 0.2 |  |
|  |  |  |  | COH: –1.9 ± 0.4 |  |
| *Idotea* spp. | den df | 85 | 13 |  |  |
|  | F |  | 0.641 |  |  |
|  | p |  | 0.602 |  |  |
|  | slope (mean ± SE) |  |  |  |  |
| *Jaera albifrons* | den df | 81 | 13 | 81 | 81 |
|  | F |  | 5.6 | 13.2 | 5.2 |
|  | p |  | 0.011 | 0.001 | 0.002 |
|  | slope (mean ± SE) |  |  | RF: –0.1 ± 0.0 |  |
|  |  |  |  | COH: –0.3 ± 0.0 |  |
