# Supplementary material for: Ecological Stoichiometry and Density Responses of Plant-Arthropod Communities on Cormorant Nesting Islands
Source: PLoS One. 2013 Apr 23;8(4):e61772. doi: 10.1371/journal.pone.0061772 (PMC3634001; doi:10.1371/journal.pone.0061772)
Supplement: Table S2 — ANOVA table of analysis of plant elemental ratios as function of island category. Shown are mean ± SE for the four island categories (reference islands (RF), abandoned cormorant island (AB), active cormorant island with low (COL) and high (COH) nest density. (DOCX) [file pone.0061772.s002.docx]

**Table S2**

| Taxa | df | F | p | Adj. R^2^ | RF | | | AB | COL | | COH | | |
| --- | --- | --- | --- | --- | --- | --- | --- | --- | --- | --- | --- | --- | --- |
| **N:C** |  |  |  |  | |  |  | | |  | |  |  |
| Herb layer (herbs and grases) | 3, 14 | **39.7** | **< 0.001** | 87 | | 0.050  ± 0.004  a | 0.057  ± 0.008  a | | | 0.084  ± 0.007  b | | 0.131  ± 0.008  c |  |
| *Alnus glutinosa* | 3, 7 | ***4.0*** | ***0.059*** | 47 | | 0.054  ± 0.010  a | 0.068  ± 0.018  a | | | 0.082  ± 0.018 a | | 0.115  ± 0.018  b |  |
| *Juniperus communis* | 1, 4 | **20.7** | **0.010** | 79 | | 0.028  ± 0.005  a |  | | | 0.060  ± 0.007  b | |  |  |
| *Sorbus aucuparia* | 3,5 | **49.6** | **< 0.001** | 94 | | 0.036  ± 0.004  a | 0.051 ± 0.007  ab | | | 0.078  ± 0.007  bc | | 0.119  ± 0.007  c |  |
| *Tanacetum vulgare* | 3, 12 | **13.3** | **< 0.001** | 71 | | 0.060  ± 0.009  a | 0.055 ± 0.016  a | | | 0.088 ± 0.014  a | | 0.153  ± 0.016  b |  |
| Poaceae | 3, 13 | **12.6** | **< 0.001** | 68 | | 0.047  ± 0.006  a | 0.064  ± 0.011 ab | | | 0.089  ± 0.009  cb | | 0.108  ± 0.011  b |  |
| **P:C** |  |  |  |  | |  |  | | |  | |  |  |
| Herb layer (herbs and grases) | 3, 13 | **6.4** | **0.007** | 50 | | 0.005  ± 0.001  a | 0.012  ± 0.002  b | | | 0.011  ± 0.002  ab | | 0.014  ± 0.002  b |  |
| *Alnus glutinosa* | 3, 5 | **7.0** | **0.031** | 69 | | 0.003  ± 0.001  a | 0.004  ± 0.001  ab | | | 0.008  ± 0.001  ab | | 0.010  0.001  b |  |
| *Tanacetum vulgare* | 3, 11 | ***3.5*** | ***0.052*** | 35 | | 0.006  ± 0.002  a | 0.014 ±  0.004  a | | | 0.013  ± 0.003  a | | 0.018  ± 0.004  b |  |
| Poaceae | 3, 11 | **5.7** | **0.013** | 50 | | 0.004  ± 0.001  a | 0.010  ± 0.002  b | | | 0.008  ± 0.002  ab | | 0.011  ± 0.002  bc |  |
| **N:P** |  |  |  |  | |  |  | | |  | |  |  |
| Herb layer (herbs and grases) | 3, 13 | **4.1** | **0.029** | 37 | | 11.5  ± 1.2  a | 4.7  ± 2.0  b | | | 8.4  ± 1.7  ab | | 10.4  ± 2.0  ab |  |
| *Alnus glutinosa* | 3, 5 | 3.2 | 0.124 | 45 | | 22.7  ± 3.0  a | 16.0  ± 4.8  a | | | 9.7  ± 4.8  a | | 11.3  ± 4.8  a |  |
| *Tanacetum vulgare* | 3, 11 | **6.8** | **0.007** | 55 | | 11.0  ± 1.1  a | 3.8  ± 1.7  b | | | 6.7  ± 1.6  ab | | 9.9  ± 1.7  a |  |
| Poaceae | 3, 11 | ***3.4*** | ***0.058*** | 33 | | 13.1  ± 1.2  ac | 6.8  ± 2.0  b | | | 10.7  ± 1.8  bc | | 10.6  ± 2.0  bc |  |
