# Supplementary material for: Ecological Stoichiometry and Density Responses of Plant-Arthropod Communities on Cormorant Nesting Islands
Source: PLoS One. 2013 Apr 23;8(4):e61772. doi: 10.1371/journal.pone.0061772 (PMC3634001; doi:10.1371/journal.pone.0061772)
Supplement: Table S3 — Statistic summary for regressions between soil and plant %N and %P. (DOCX) [file pone.0061772.s003.docx]

**Table S3**

| taxa | %N | | | | %P | | | |
| --- | --- | --- | --- | --- | --- | --- | --- | --- |
|  | df | F | p | slope | df | F | p | slope |
| *Alnus glutinosa* | 8 | **12.9** | **0.008** | 0.15 | 7 | **9.3** | **0.019** | 0.30 |
| *Juniperus communis* | 4 | **11.0** | **0.030** | 0.21 |  |  |  |  |
| *Sorbus aucuparia* | 6 | **14.5** | **0.009** | 0.23 |  |  |  |  |
| *Tanacetum vulgare* | 7 | **11.9** | **0.011** | 0.20 | 7 | **33.2** | **0.001** | 0.26 |
| Poaceae | 8 | **12.0** | **0.008** | 0.21 | 7 | **28.5** | **0.001** | 0.36 |
