# Supplementary material for: Ecological Stoichiometry and Density Responses of Plant-Arthropod Communities on Cormorant Nesting Islands
Source: PLoS One. 2013 Apr 23;8(4):e61772. doi: 10.1371/journal.pone.0061772 (PMC3634001; doi:10.1371/journal.pone.0061772)
Supplement: Table S4 — Results of linear mixed effects model (lme) testing for differences in elemental ratios (mean ± SE) of brackish invertebrates between reference islands (RF) and cormorant islands (abandoned (AB) and active cormorant islands with low and (COL) high (COH) nest density). Shown are the number of samples (n), number of islands (islands), F- and p- values from ANOVA for lme, the slope for wave exposure, and mean ± SE for the four island categories.* indicate significant difference (p<0.05), • marginal significant difference (p<0.1) from reference islands. (DOCX) [file pone.0061772.s004.docx]

**Table S4**

| **Taxa** | **n** | **is** | **is** | **p** | **Slope**  **(mean ± SE)** | **Island category** | **Mean ± SE** |
| --- | --- | --- | --- | --- | --- | --- | --- |
| **N:C** |  |  |  |  |  |  |  |
| ***Fucus vesiculosus*** | 102 | 17 | 17 |  |  | RF | 0.035 ± 0.003 |
| Island category (df = 3) |  |  |  | 0.033 |  | AB | 0.025 ± 0.003 |
|  |  |  |  |  |  | COL | 0.035 ± 0.008 |
|  |  |  |  |  |  | COH | 0.055 ± 0.004* |
| **Epiphytic algae** | 154 | 17 | 17 |  |  | RF | 0.054 ± 0.006 |
| Island category (df = 3) |  |  |  | 0.023 |  | AB | 0.042 ± 0.002 |
| Wave exposure (df = 1) |  |  |  | 0.015 | -0.006 ± 0.002 | COL | 0.065 ± 0.004 |
|  |  |  |  |  |  | COH | 0.082 ± 0.003** |
| **P:C** |  |  |  |  |  |  |  |
| ***Fucus vesiculosus*** | 33 | 17 | 17 |  |  | RF | 1.23e-3 ± 0.08e-3 |
| Island category (df = 3) |  |  |  | 0.055 |  | AB | 1.25e-3 ± 0.08e-3 |
|  |  |  |  |  |  | COL | 1.68e- 3 ± 0.34e-3• |
|  |  |  |  |  |  | COH | 1.86e- 3 ± 0.17e-3* |
| **Epiphytic algae** | 34 | 16 | 16 |  |  | RF | 4.01e- 3 ± 0.37e-3 |
| Island category (df = 3) |  |  |  | 0.063 |  | AB | 4.50e- 3 ± 0.61e-3 |
|  |  |  |  |  |  | COL | 4.85e- 3 ± 0.42e-3 |
|  |  |  |  |  |  | COH | 5.87e- 3 ± 0.39e-3* |
| **N:P** |  |  |  |  |  |  |  |
| ***Fucus vesiculosus*** | 33 | 17 | 17 |  |  | RF | 28.01 ± 2.69 |
| Island category (df = 3) |  |  |  | 0.999 |  | AB | 28.48 ± 1.74 |
|  |  |  |  |  |  | COL | 27.52 ± 6.37 |
|  |  |  |  |  |  | COH | 28.10 ± 2.56 |
| **Epiphytic algae** | 24 | 16 | 16 |  |  | RF | 14.3 ± 1.45 |
| Island category (df = 3) |  |  |  | 0.255 |  | AB | 8.68 ± 1.71 |
|  |  |  |  |  |  | COL | 11.99 ± 0.98 |
|  |  |  |  |  |  | COH | 13.92 ± 0.79 |
