# Supplementary material for: Ecological Stoichiometry and Density Responses of Plant-Arthropod Communities on Cormorant Nesting Islands
Source: PLoS One. 2013 Apr 23;8(4):e61772. doi: 10.1371/journal.pone.0061772 (PMC3634001; doi:10.1371/journal.pone.0061772)
Supplement: Table S5 — Results of linear mixed effects model (lme) testing for differences in elemental ratios (mean ± SE) of terrestrial arthropods and insects between reference islands (RF) and cormorant islands (abandoned (AB) and active (AC)). * indicate significant difference (in bold) (p<0.05) from reference islands. (DOCX) [file pone.0061772.s005.docx]

**Table S5**

| **Taxa** | **Island category** | **N:C** | **P:C** | **N:P** |
| --- | --- | --- | --- | --- |
| **Herbivores** | RF | 0.195 ± 0.005 | 0.016 ± 0.001 | 0.016 ± 0.001 |
|  | AB | 0.197 ± 0.012 | 0.016 ± 0.002 | 0.016 ± 0.002 |
|  | AC | 0.198 ± 0.006 | 0.019 ± 0.001 | 0.019 ± 0.001 |
| Aphidina | RF | 0.112 ± 0.012 |  |  |
|  | AB |  |  |  |
|  | AC | 0.140 ± 0.017 |  |  |
| Cercopidea | RF | 0.210 ± 0.006 | 0.016 ± 0.001 | 0.016 ± 0.001 |
|  | AB | 0.200 ± 0.006 | 0.013 ± 0.000* | 0.013 ± 0.000* |
|  | AC | 0.95 ± 0.008 | 0.016 ± 0.001 | 0.016 ± 0.001 |
| Lepidoptera larvae | RF | 0.221 ± 0.019 | 0.020 ± 0.004 | 0.020 ± 0.004 |
|  | AB | 0.254 ± 0.038 | 0.025 ± 0.004 | 0.025 ± 0.004 |
|  | AC | 0.221 ± 0.012 | 0.026 ± 0.002 | 0.026 ± 0.002 |
| Chrysomelidae | RF | 0.169 ± 0.010 | 0.015± 0.001 | 0.015± 0.001 |
|  | AB | 0.174 ± 0.017 | 0.016 ± 0.005 | 0.016 ± 0.005 |
|  | AC | 0.176 ± 0.016 | 0.015 ± 0.001 | 0.015 ± 0.001 |
| Curculionidae | RF | 0.180 ± 0.009 | 0.011± 0.001 | 0.011± 0.001 |
|  | AB | 0.148 ± 0.009* | 0.010 ± 0.002 | 0.010 ± 0.002 |
|  | AC | 0.195 ± 0.010 | 0.014 ± 0.000 | 0.014 ± 0.000 |
| **Detritivore** | RF | 0.217 ± 0.020 | 0.041 ± 0.003 | 0.041 ± 0.003 |
|  | AB | 0.210 ± 0.016 | 0.037 ± 0.002 | 0.037 ± 0.002 |
|  | AC | 0.223 ± 0.016 | 0.046 ± 0.006 | 0.046 ± 0.006 |
| Isopoda | RF | 0.189 ± 0.002 | 0.041 ± 0.003 | 0.041 ± 0.003 |
|  | AB | 0.185 ± 0.006 | 0.037 ± 0.002 | 0.037 ± 0.002 |
|  | AC | 0.184 ± 0.005 | 0.046 ± 0.006 | 0.046 ± 0.006 |
| Collembola | RF | 0.263 ± 0.003 |  |  |
|  | AB | 0.259 ± 0.002 |  |  |
|  | AC | 0.286 ± 0.008* |  |  |
| Brachycerid diptera | RF | 0.203 ± 0.012 |  |  |
|  | AB | 0.232 ± 0.024 |  |  |
|  | AC | 0.189 ± 0.013 |  |  |
| **Chironomidae** | RF | 0.233 ± 0.003 | 0.020 ± 0.001 | 0.020 ± 0.001 |
|  | AB | 0.233 ± 0.005 | 0.018 ± 0.001 | 0.018 ± 0.001 |
|  | AC | 0.219 ± 0.005* | 0.021 ± 0.000 | 0.021 ± 0.000 |
| **Predators** | RF | 0.22 ± 0.01 | 0.017 ± 0.002 | 0.017 ± 0.002 |
|  | AB | 0.24 ± 0.01 | 0.017 ± 0.003 | 0.017 ± 0.003 |
|  | AC | 0.23 ± 0.00 | 0.019 ± 0.007 | 0.019 ± 0.007 |
| Araneidae | RF | 0.264 ± 0.002 | 0.017 ± 0.001 | 0.017 ± 0.001 |
|  | AB | 0.263 ± 0.004 | 0.016 ± 0.003 | 0.016 ± 0.003 |
|  | AC | 0.264 ± 0.007 | 0.019 ± 0.003 | 0.019 ± 0.003 |
| Linyphiidae | RF | 0.241 ± 0.006 | 0.019 ± 0.003 | 0.019 ± 0.003 |
|  | AB | 0.229 ± 0.004 | 0.020 ± 0.001 | 0.020 ± 0.001 |
|  | AC | 0.221 ± 0.004* | 0.021 ± 0.001 | 0.021 ± 0.001 |
| Tetragnathidae | RF | 0.259 ± 0.004 |  |  |
|  | AB | 0.261 ± 0.003 |  |  |
|  | AC | 0.260 ± 0.006 |  |  |
| Lycosidae | RF | 0.264 ± 0.002 |  |  |
|  | AB | 0.277 ± 0.009 |  |  |
|  | AC | 0.252 ± 0.000 |  |  |
| Coccinellidae | RF | 0.210 ± 0.006 | 0.015 ± 0.001 | 0.015 ± 0.001 |
|  | AB | 0.238 ± 0.044 | 0.014 ± 0.000 | 0.014 ± 0.000 |
|  | AC | 0.231 ± 0.009 | 0.016 ± 0.003 | 0.016 ± 0.003 |
| Carabidae | RF | 0.205 ± 0.003 | 0.012 ± 0.001 | 0.012 ± 0.001 |
|  | AB | 0.213 ± 0.004 | 0.012 ± 0.001 | 0.012 ± 0.001 |
|  | AC | 0.205 ± 0.004 | 0.013 | 0.013 |
| Staphylinidae | RF | 0.195 ± 0.011 |  |  |
|  | AB | 0.226 ± 0.022 |  |  |
|  | AC | 0.233 ± 0.010 |  |  |
| *Nabis* spp. (Nabidae) | RF | 0.193 ± 0.008 |  |  |
|  | AB | 0.195 ± 0.013 |  |  |
|  | AC | 0.204 ± 0.015 |  |  |
| Formicidae | RF | 0.232 ± 0.008 |  |  |
|  | AB | 0.247 ± 0.004 |  |  |
|  | AC | 0.232 ± 0.009 |  |  |
