# Supplementary material for: Ecological Stoichiometry and Density Responses of Plant-Arthropod Communities on Cormorant Nesting Islands
Source: PLoS One. 2013 Apr 23;8(4):e61772. doi: 10.1371/journal.pone.0061772 (PMC3634001; doi:10.1371/journal.pone.0061772)
Supplement: Table S6 — Summary statistic for regressions between resources and consumer N:C, P:C, and N:P. Slopes provided are adjusted slopes, that is adjusted slopes are equal to the calculated slopes if regressions were significant (in bold); slopes from insignificant regressions (p>0.05) were set to zero. (DOCX) [file pone.0061772.s006.docx]

**Table S6**

| **Taxa** | **N:C** | | | | | | **P:C** | | | **N:P** | | | | | |  |
| --- | --- | --- | --- | --- | --- | --- | --- | --- | --- | --- | --- | --- | --- | --- | --- | --- |
|  | df | | | *F* | Slope | | df | *F* | Slope | df | *F* | | Slope | | |  |
| **Terrestrial arthropods** | | | | | | | | | | | | | | |  |  |
| Herbivores*^p^ | |  | |  | |  |  |  |  |  | |  | |  | | |
| Aphidina | | 3 | | 0.4 | | 0 |  |  |  |  | |  | |  | | |
| Cercopidea | | 12 | | 0.7 | | 0 | 11 | 1.8 | 0 | 11 | | 1.5 | | 0 | | |
| Lepidoptera larvae | | 13 | | 0.3 | | 0 | 13 | 2.9 | 0 | 13 | | 0.2 | | 0 | | |
| Chrysomelidae | | 8 | | 1.1 | | 0 | 12 | 0.1 | 0 | 12 | | 0.1 | | 0 | | |
| Curculionidae | | 5 | | 1.0 | | 0 | 9 | 0.0 | 0 | 9 | | 0.4 | | 0 | | |
| Detritivores*^p^ | |  | |  | |  |  |  |  |  | |  | |  | | |
| Isopoda | | 12 | | 0.2 | | 0 | 12 | 0.0 | 0 | 12 | | 0.1 | | 0 | | |
| Collembola | | **8** | | **10.4^*^** | | **0.10** |  |  |  |  | |  | |  | | |
| Predators | |  | |  | |  |  |  |  |  | |  | |  | | |
| Araneidae*^ch^ | | 13 | | 1.8 | | 0 | 10 | 4.3 | 0 | 10 | | 0.5 | | 0 | | |
| Linyphiidae *^ch^ | | 12 | | 4.4 | | 0 | 8 | 3.9 | 0 | 8 | | 0.9 | | 0 | | |
| Tetragnathidae*^ch^ | | 9 | | 0.7 | | 0 |  |  |  |  | |  | |  | | |
| Lycosidae*^ch^ | | 8 | | 4.9 | | 0 |  |  |  |  | |  | |  | | |
| Carabidae*^col^ | | 8 | | 0.3 | | 0 |  |  |  |  | |  | |  | | |
| **Brackish invertebrates *^3^** | | | | | |  |  |  |  |  | |  | |  | | |
| Chironomidae *^ep^ | | | 16 | 2.2 | | 0 | 13 | 0.2 | 0 | **13** | | **12.2^**^** | | **-0.79** | | |
| *Gammarus* spp. *^ep^ | | | 16 | 2.9 | | 0 | 15 | 0.8 | 0 | **15** | | **9.0^**^** | | **0.78** | | |
| *Idotea* spp. *^ep^ | | | 16 | 2.2 | | 0 | 13 | 0.3 | 0 | 13 | | 0.1 | | 0 | | |
| *Idotea* spp. *^fu^ | | | **16** | **10.0^*^** | | **0.25** | 14 | 0.1 | 0 | 14 | | 0.7 | | 0 | | |
| *Jaera albifrons* *^ep^ | | | 13 | 2.6 | | 0 |  |  |  |  | |  | |  | | |
| *Theodoxus fluviatilis* ^*ep^ | | | 14 | 4.4 | | 0 |  |  |  |  | |  | |  | | |

**Resources:** *^p^ terrestrial plants, *^col^ Collembola, *^ch^ Chironomidae; * ^ep^ Epiphytic algae**,** *^fu^ *F****.*** *vesiculosus***,**
