# Supplementary material for: Ecological Stoichiometry and Density Responses of Plant-Arthropod Communities on Cormorant Nesting Islands
Source: PLoS One. 2013 Apr 23;8(4):e61772. doi: 10.1371/journal.pone.0061772 (PMC3634001; doi:10.1371/journal.pone.0061772)
Supplement: Table S7 — Results of linear regressions (lm) and generalized linear models (glm) testing for a linear relationship between plant quality (leaf N:C and P:C-content) and plant quantity (aboveground plant biomass (g/62.5 cm2) and arthropod densities. Shown are the direction of effect positive (+) and negative (−). Shown are only significant results (p<0.05). (DOCX) [file pone.0061772.s007.docx]

**Table S7**

| **Taxa** | **Model** | **Vegetation cover** | **Model** | **Plant species richness** |
| --- | --- | --- | --- | --- |
| **Herbivores** |  |  |  |  |
| Aphidina | lm | (+) | lm |  |
|  |  | F = 5.0, p = 0.041, |  |  |
|  |  | df =15 , R2= 20.0% |  |  |
| Cercopidea | glm |  | glm |  |
| Lepidoptera larvae*1 | glm | (-) | glm | (-) |
|  |  | t = -1.9, p = 0.074, |  | F = 4.0, p = 0.063, |
|  |  | P(χ2) = 0.066, df =15 |  | df =15 , R2= 15.9% |
| Chrysomelidae | lm | (+) |  |  |
|  |  | F = 20.9, p < 0.001, | lm |  |
|  |  | df =15 , R2= 55.4% |  |  |
| Curculionidae | lm | (+) | lm |  |
|  |  | F = 17.3, p < 0.001, |  |  |
|  |  | df =15, R2= 50.5% |  |  |
| Herbivorous Heteroptera | lm |  | lm |  |
| **Detritivore** |  |  |  |  |
| Isopoda | glm |  | glm |  |
| Collembola | lm | (+) | lm | (+) |
|  |  | F = 27, p < 0.0001, |  | F = 12.7, p = 0.003, |
|  |  | df =15, R2= 61.9% |  | df =15, R2= 41.1% |
| Brachycerid diptera | lm | (-) | lm | (-) |
|  |  | F = 13.7, p < 0.002, |  | F = 4.7, p = 0.047, |
|  |  | df =15, R2= 44.2% |  | df =15, R2= 18.8% |
| ***Chironomidae*** | lm | (-) | lm | (-) |
|  |  | F = 8.7, p = 0.010, |  | F = 5.6, p = 0.032, |
|  |  | df=15, R2= 32.5% |  | df =15, R2= 22.3% |
| **Predators** |  |  |  |  |
| Araneidae | lm |  | lm |  |
| Linyphiidae | lm |  | lm |  |
| Tetragnathidae | glm |  | glm |  |
| Pachygnatha | lm |  | lm |  |
| Lycosidae | glm | (-) | glm |  |
|  |  | t = -2.8,p = 0.015, |  |  |
|  |  | P(χ2) < 0.002, df =15 |  |  |
| Coccinellidae | lm |  | lm |  |
| Carabidae | lm | (+) | lm |  |
|  |  | F = 9.5, p = 0.007, |  |  |
|  |  | df =15 , R2= 34.8% |  |  |
| Staphylinidae | glm |  | glm |  |
| Nabis spp. (Nabidae) | glm |  | glm |  |
| Formicidae | lm |  | lm |  |
| Parasitic hymenoptera | lm |  | lm |  |
